# Scheduling truck arrivals for efficient container flow management in port logistics

**DOI:** 10.1007/s10100-025-00976-x

**Published:** 2025-03-21

**Authors:** Daniil Baldouski, Miklós Krész, Balázs Dávid

**Affiliations:** 1 University of Primorska, Titov trg 4, 6000 Koper, Slovenia; 2 InnoRenew CoE, Livade 6a, 6310 Izola, Slovenia; 3 University of Szeged, 13 Dugonics square, Szeged, 6720 Hungary

**Keywords:** Scheduling, Port logistics, Container flow optimization, Simulation, Mixed-integer linear programming

## Abstract

The management of truck arrivals at container terminals is crucial for efficient port operations. Congestions developing both outside and inside the gates can cause logistical problems, while also having a significant impact on the environment and the surroundings of the port. Therefore, optimizing truck queues outside the gates of the port, as well as routing of trucks inside the terminals can lead to an improvement in the overall efficiency of the port processes. This paper presents a mixed-integer linear programming formulation to determine these optimal truck routes and schedules. The model considers a port with an external parking lot, multiple gates, internal roadways, and docks. A rolling horizon heuristic is also developed for the solution of instances where the model is otherwise intractable. The developed methods are evaluated on instances simulated based on real-world data.

## Introduction

Efficient port operations are crucial for international trade, as container terminals serve as significant nodes within global supply chain networks. The rapid expansion of container trade, with a significant average annual increase in volume (Gharehgozli et al. 2016), has placed an emphasis on optimizing these critical points. According to Carlo et al. (2014), internal transport operations play an important role in connecting the seaside, yard side, and landside areas of container terminals. These operations, coupled with the dynamic needs of global commerce, present unique challenges and opportunities for operational optimization.

There are extensive studies in marine container terminal modeling, particularly in the implementation of operational efficiencies within port logistics. Studies have explored various aspects of port operations, including ship routes and loading plans (Korsvik et al. 2010; Monaco et al. 2014), berth and crane allocation (Bierwirth and Meisel 2010; Meisel and Bierwirth 2013; Kizilay and Eliiyi 2021), yard tracks (Stojaković and Twrdy 2021), drayage operations (Huynh and Walton 2011), as well as truck scheduling and container storage (Angeloudis and Bell 2010; Chen et al. 2013; Gharehgozli et al. 2014; Carlo et al. 2014). These complexities, introduced by economic fluctuations, geographical disruptions, evolving market demands and environmental concerns (Raeesi et al. 2023), require innovative approaches in terminal operations, material handling, and overall logistical coordination.

Stahlbock and Voß (2008) and Steenken et al. (2004) provide a comprehensive review of over 300 literature sources, highlighting the critical role of integrated scheduling systems in improving terminal operations. The impact of such systems is further examined by Huynh and Walton (2008), who investigated the operational limits on truck entries and the consequential effects on truck turn time and yard crane utilization. Similarly, Namboothiri and Erere (2006); Namboothiri and Erera (2008) analyzed how port appointment-based access control systems influence the management of truck fleets providing container pickup and delivery services. This corresponds to our model’s focus on optimizing container flow and truck scheduling within ports. Limited maneuver space, strict loading and unloading procedures, and traffic restrictions on internal roads further complicate this process (Expósito-Izquierdo et al. 2017). Additional insights into truck appointment systems (TAS) can be found in recent studies that explore various dimensions of these systems (Zehendner and Feillet 2015; Ambrosino and Caballini 2015; Ambrosino and Peirano 2016). In 2015, Phan and Kim (2015) proposed a new TAS that, unlike conventional approaches, includes a negotiation mechanism to balance the competing demands of container terminals and trucking companies. Then, in 2019 (Yi et al. 2019), authors continued and described a non-linear mathematical model of this approach and analyzed the performance of the model using practical data for truck arrivals.

This paper presents a mixed-integer linear programming (MILP) model to improve container movement within ports. Our model integrates the scheduling of ships, trucks, gates, docks, and internal roads. The adaptability of the MILP model allows us to accommodate various port layouts and traffic patterns, making it versatile across different scenarios. The goal of the model is to minimize both the delays and the operational costs. It is possible for our model to be extended to incorporate multiple objectives, such as truck emissions, energy consumption, waiting queue time, truck turnaround (Abdelmagid et al. 2022a) and more (Abdelmagid et al. 2022b), and perform multiobjective analysis in order to explore more realistic scenarios. However, the absence of real-world truck arrivals datasets, especially detailing individual truck arrivals, remains a significant obstacle to implementing this approach.

To our best knowledge, there are no academic literature developing an exact mathematical model that integrates the entire terminal network through a comprehensive set of constraints that enforce at the same time:

- FIFO rules on all road segments,
- Maximum capacity limits for all the roads and facilities,
- Processing time requirements at gates and docks,
- Precedence relationships between trucks during the entire path,
- Coordination with ship schedules and dock assignments.

Moreover, we adopt a continuous approach, meaning that we have independent from the context time units, which could be easily parametrized at the input stage to better satisfy the practical needs of the specific port environment. This represents a difference from traditional TAS implementations that typically focus primarily on gate congestion through discrete time window allocation, while the goal of our model is to find an overall optimal solution.

This work is based on the previous model for port container flow optimization presented at the SOR 23 conference (Baldouski et al. 2023). The refined model is more streamlined and is capable of solving larger and more complex instances. The new model is extended with precedence and ordering constraints to enforce the correct sequence of operations. Additionally, we removed unnecessary constraints covering container loading since this part is now considered a separate problem and is presented in a simplified way. The ability of the improved model to solve large instances relies heavily on the newly added rolling horizon heuristic, which is described in Sect. 4. We demonstrate the ability of the new model to improve efficiency compared to the previous model by using more exhaustive tests, the results of which are presented in Sect. 5.

The outline of the paper is the following: first, the description of the problem (Sect. 2), which summarizes the main challenges and constraints in port logistics that our model aims to address. Then in the mathematical model section (Sect. 3), we detail the components of our mixed-integer linear programming model, including variables, constraints, and the objective function. The rolling horizon heuristic for the solution of the problem is introduced in Sect. 4. In Sect. 5 we present the application of our model to simulated instances based on the Port of Koper and demonstrate its effectiveness with potential scalability. We conclude the article by summarizing the key findings and suggesting directions for future research to further improve port operations efficiency.

## Problem description

The objective of our study is to improve port logistics efficiency by optimizing container flow. Each truck carries one container, and the containers are loaded onto ships at the dock using a crane. Constraints in this process include FIFO rules on all roads, one-way directionality, facilities such as gates and docks can process only one truck at a time, an external parking lot, and each road has its own maximum truck capacity. Container loading onto ships is mainly considered a separate problem and is simplified as a result.

The port infrastructure is defined as follows:

- **Gates:** the port has a specified number of gates that serve as entry points for trucks.
- **Docks:** there are a fixed number of docks where ships are berthed for container unloading from trucks and loading onto ships.
- **External parking lot:** trucks initially wait in an external parking lot with limited capacity.
- **Roads:** a network of roads connects the parking lot, gates, and docks. Each road segment has a specific capacity and trucks operate on a FIFO basis.
- **Processing times:** various operations within the port have associated processing times, including:
  - **Gate processing:** the time required for a truck to pass through a gate.
  - **Container unloading:** the time required to unload a container from a truck at the dock.
  - **Container loading:** the time required to load a container onto a ship.
  - **Travel times:** the time required for trucks to travel between the parking lot and gates or between gates and docks.

**Fig. 1:**
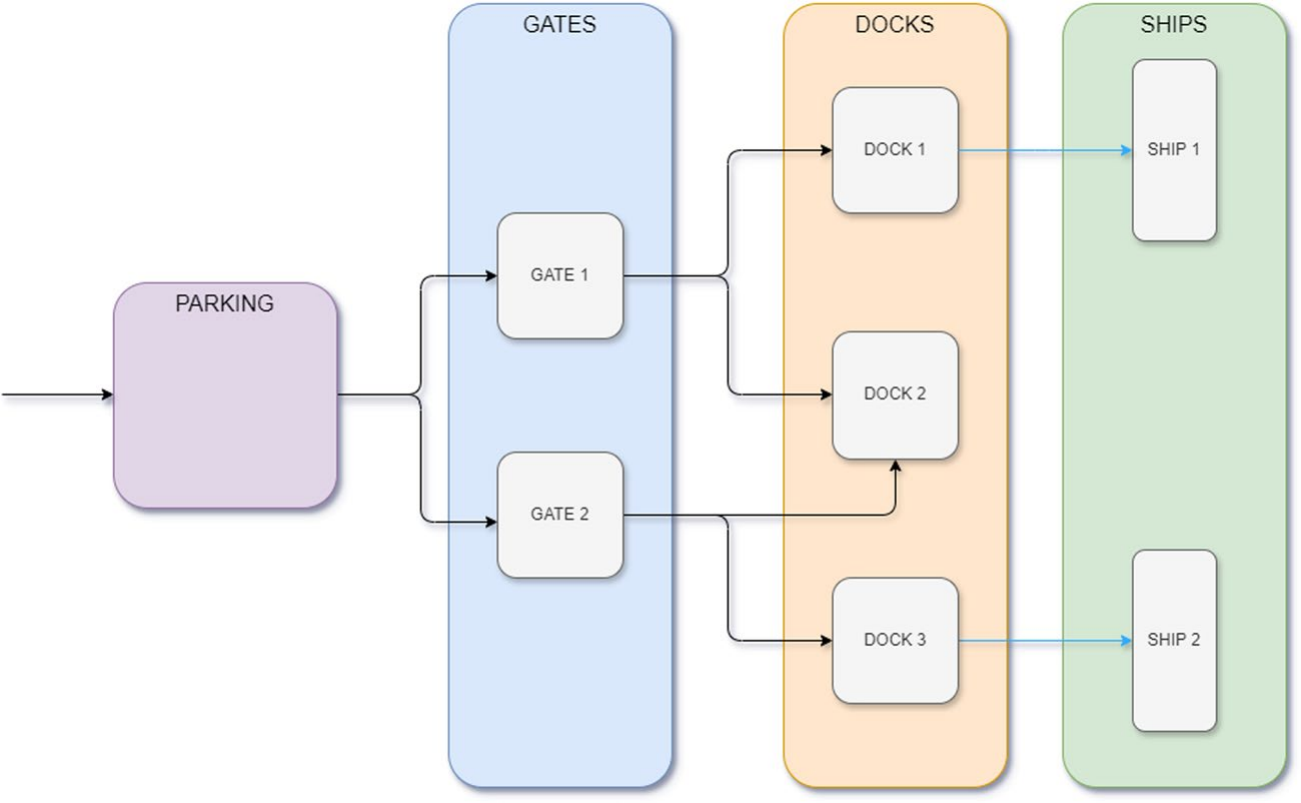
Example of a port structure with 2 gates, 3 docks

An example of the entire port process is shown in Fig. 1, which illustrates the flow of containers through the various stages. In this example, the port consists of 2 gates, 3 docks, and there are road connections from gate 1 to docks 1 and 2 and from gate 2 to docks 2 and 3. There are 2 ships that are currently at the docks 1 and 3.

The port operates on a continuous basis, where the movement of trucks begins as soon as possible and continues under specific constraints.

- **FIFO:** all roads operate on a First-In, First-Out (FIFO) principle.
- **One-way traffic:** all roads have designated directions (from parking to gates or from gates to docks).
- **Facility capacity:** gates and docks can process only one truck at a given time.
- **Road capacity:** each road segment has a maximum capacity, limiting the number of trucks it can hold simultaneously.

The process for the trucks follows these steps:

1. **Arrival and parking:** trucks arrive at the port and queue in the external parking lot.
2. **Gate entry:** trucks enter the port through a designated gate.
3. **Dock entry and container unloading:** trucks are assigned to a specific dock to which they come from designated gates and unload their containers there. After container unloading, trucks are considered removed from the system, and only their containers remain.
4. **Loading onto ships:** containers are loaded onto their designated ships in a simplified way.

We assume that trucks are assigned to ships in advance. In addition, the ship arrival times and their dock assignments are known in advance as well. This means that in the preprocessing step, trucks can be assigned to their docks as well, which is why such truck-dock assignment is considered as a constant parameter of the problem that is given in advance.

The goal of the model is to optimize container flow by assigning trucks to specific gates and choosing their arrival and departure times for each entity within the port environment (parking lot, gates, docks and all the roads in-between). The objective is to minimize the overall costs, based on the time required for the trucks to complete their tasks within the port, starting from their earliest possible arrival to the parking lot to the unloading of their containers in docks (which is referred to as departure from docks throughout this paper).

We propose a mixed-integer linear programming model (MILP) that accommodates these complexities. The model allows the system to adapt to various demands and is generalizable under different port structure configurations.

## Mathematical model

The mathematical model developed for port logistics optimization requires a well-defined set of input data to function. This section presents a formal representation of the parameters necessary for describing the operational structure and constraints within the port environment. These parameters incorporate various aspects of truck scheduling such as:

- **Facilities**: gates, docks, and their connectivity.
- **Transport**: trucks and ships, including associated cost and time-related metrics.
- **Constraints**: operational limitations and capacities.

Time related parameters and variables are measured in generalizable integer valued time units. However, in practice, the time variables for our port structure and the simulated truck instances (see Sect. 5) are measured in minutes for simplicity reasons.

### Problem data

The problem data are formally represented as follows:

*V* is the set of all vehicles (trucks), *S* is the set of all ships. Port structure is defined by the set of gates *G* and docks *D*.

Then for each truck $v \in V$, gate $g \in G$, dock $d \in D$, ship $s \in S$, we establish the following parameters:

1. **Assignment parameters:**
  $aVS(v, s) = 1$ if truck *v* and the ship *s* are assigned to each other, 0 otherwise.
  *aSD*(*s*) is the index of the dock to which ship *s* is assigned.
  *aVD*(*v*) is the index of the dock to which truck *v* is assigned to.
  $aGD(g, d) = 1$ if gate *g* and the dock *d* are connected to each other with a road, 0 otherwise.
2. **Processing time parameters:**
  *tVG*(*v*, *g*) - time required to process truck *v* at gate *g*.
  *tVD*(*v*) - time required to process truck *v* at the dock.
  *tVS*(*v*) - time required to load the truck container *v* onto the ship.
3. **Travel time parameters:**
  *tG*(*g*) - travel time from the parking lot to the gate *g*.
  *tGD*(*g*, *d*) - travel time from the gate *g* to the dock *d*.
4. **Capacity parameters:**
  *cP* - capacity of the parking lot.
  *cG*(*g*) - capacity of the road from the parking lot to gate *g*.
  *cGD*(*g*, *d*) - capacity of the road from gate *g* to dock *d*.
5. **Scheduling parameters:**
  *pV*(*v*) - earliest possible arrival time of truck *v* to the parking lot.
  *eV*(*v*) - latest possible departure time of truck *v*.
  *pS*(*s*) - earliest possible arrival time of ship *s*.
  *eS*(*s*) - latest possible departure time of ship *s*.
6. **Cost parameters:**
  *uV*(*v*) - unit cost of the use of truck *v*.

### Variables

Similar to the parameters of the model, variables can be grouped into three main categories: assignment, time and auxiliary.

#### Assignment variables

These are **binary** variables that denote the assignment of specific tasks within the operational structure of the port, such as the allocation of trucks to specific gates and docks. For each truck $v \in V$ and gate $g \in G$ we define the following variable:

- $x_{v, g} = 1$ if the truck *v* and the gate *g* are assigned to each other.

#### Time variables

The variables in this category are **continuous** and represent different aspects of time related to truck movement and processing within the port, including arrival and departure times at various points in the port logistics chain.

1. **Trucks:**
  For each truck $v \in V$, gate $g \in G$ we define the following variables:
  $pa_{v}$ - arrival time of the truck *v* in the parking lot.
  $pd_{v}$ - departure time of the truck *v* from the parking lot.
  $ga_{v, g}$ - arrival time of the truck *v* at the gate *g*.
  $gd_{v, g}$ - departure time of the truck *v* from the gate *g*.
  $da_{v}$ - arrival time of the truck *v* at the dock.
  $dd_{v}$ - departure time of the truck *v* from the dock.
2. **Ships:**
  For each ship $s \in S$ and dock $d \in D$ we define the following variables:
  $sa_{s}$ - arrival time of the ship *s* at the dock.
  $sd_{s}$ - departure time of the ship *s* from the dock.

#### Auxiliary variables

These are **binary** variables that help with the handling of complex constraints and are used for the purposes of linearization. Most of them define a precedence relation between two different trucks (e.g. $\alpha _{v, w} = 1$ if the arrival time of truck *w* at the parking lot occurs before the arrival time at the parking lot of truck *v*), one helps with the precedence constraint of ships, and some represent a specific interaction between pairs of different trucks. All of them are defined in the following Sect. 3.3 together with their respective constraints.

For each truck $v, w \in V$, such that $v \ne w$ and gate $g \in G$ we define the following variables:

- *Precedence between trucks at facilities (gates, docks) and the parking lot:*
  - $$\alpha _{v, w}, \beta _{v, w}, \delta _{v, w}, \theta _{v, w}, \mu _{v, w}, \nu _{v, w}, \sigma _{v, w, g}, \kappa _{v, w}$$.
- *Precedence between trucks at the roads:*
  - $$\phi _{v, w}$$.
- *Specialized truck interactions:*
  - $$\gamma _{v, w}, \eta _{v, w}, \xi _{v, w}, \upsilon _{v, w, g}, \zeta _{v, w, g}, \omega _{v, w, g}, \tau _{v, w, g}$$.

For each ship $s \in S$, we define the following variables:

- *Precedence between ships at the docks:*
  - $$\rho _{s_1, s_2}$$.

### Constraints

Similar to the model variables, constraints can be grouped into four main categories:

- **Assignment constraints:** these ensure the correct distribution of trucks, ordering which entities (gates, docks, and the corresponding roads) are paired with which vehicles (trucks, ships).
- **Time constraints:** these control the timeline of operations, ordering when certain tasks should start or end.
- **Assignment-time constraints:** these integrate task assignment and timeline, ensuring that scheduled assignments follow the corresponding temporal limitations.
- **Capacity constraints:** these limit the volume of tasks assigned, ensuring that no entity is overloaded beyond its operational capacity.

#### Assignment constraints

- *Each truck is assigned to exactly one gate.*
  For each truck $v \in V$:
  1 $$\begin{aligned} \sum _{g \in G} x_{v, g} = 1 \end{aligned}$$
- *Trucks cannot be assigned to gates from which there is no access to the required dock.*
  For each truck $v \in V$, gate $g \in G$:
  2 $$\begin{aligned} x_{v, g} \le aGD(g, aVD(v)) \end{aligned}$$

#### Time constraints

We use ’Big M’ in our model for trucks and ships. ’Big M’ helps us deal with constraints by setting two large constants: $M_v$ for trucks and $M_s$ for ships. They are defined as following:

$$\begin{aligned} \begin{aligned} M_v&= 2 \cdot \max _{v \in V}(eV(v)) + 1, \\ M_s&= 2 \cdot \max _{s \in S}(eS(s)) + 1, \end{aligned} \end{aligned}$$

where *eV*(*v*) and *eS*(*s*) are the latest possible departure times of the trucks and ships respectively, defined in the Sect. 3.1.

- *Gates and docks can process only one truck at a time.*
  This and similar constraints are defined with the help of auxiliary variables. For this part, we introduce the auxiliary variables $\sigma _{v, w, g}, \kappa _{v, w}$ and $\rho _{s_1, s_2}$. So for each pair of trucks $v, w \in V$ such that $v \ne w$.
  - Gates - for each gate *g*:
    $\sigma _{v, w, g} = 1$ if and only if truck *v* departs from gate *g* not later than the arrival of truck *w* to the same gate *g*, that is, $gd_{v, g} \le ga_{w, g}$. Let $\sigma _{v, w, g} = 0$ otherwise, and in the case $gd_{v, g} = ga_{w, g}$ let $\sigma _{v, w, g} = 1$ and $\sigma _{w, v, g} = 0$ if $v < w$ in a lexicographic order.
    This is necessary because of edge cases, for example having $gd_{v, g} = ga_{w, g} = 0$, which is the case if both trucks *v* and *w* are not assigned to gate *g*. In this case, a lexicographic order would resolve the issue, moreover it is easily enforced in practice, since trucks most likely have a name, or a unique id linked to them.
    The above are guaranteed by the following constraints:
    3 $$\begin{aligned} \begin{aligned}&gd_{v, g} - ga_{w, g} \le M_v \cdot (1 - \sigma _{v, w, g}) \\&\sigma _{v, w, g} + \sigma _{w, v, g} = 1 \end{aligned} \end{aligned}$$
  - Docks - if $$aVD(v) = aVD(w)$$:
    $\kappa _{v, w} = 1$ if and only if $dd_{v} \le da_{w}$ and $\kappa _{v, w} = 0$ otherwise, and in the case $dd_{v} = da_{w}$ let $\kappa _{v, w} = 1$ and $\kappa _{w, v} = 0$ if $v < w$ in a lexicographic order. The above are guaranteed by the following constraints:
    4 $$\begin{aligned} \begin{aligned}&dd_{v} - da_{w} \le M_v \cdot (1 - \kappa _{v, w}) \\&\kappa _{v, w} + \kappa _{w, v} = 1 \end{aligned} \end{aligned}$$
- *Docks can process only one ship at a time.*
  For each pair of ships $s_1, s_2 \in S$ such that $s_1 \ne s_2$ and $aSD(s_1) = aSD(s_2)$:
  - $\rho _{s_1, s_2} = 1$ if and only if $sd_{s_1} \le sa_{s_2}$ and $\rho _{s_1, s_2} = 0$ otherwise, and in the case $sd_{s_1} = sa_{s_2}$ let $\rho _{s_1, s_2} = 1$ and $\rho _{s_2, s_1} = 0$ if $s_1 < s_2$ in a lexicographic order. The above are guaranteed by the following constraints:
    5 $$\begin{aligned} \begin{aligned}&sd_{s_1} - sa_{s_2} \le M_s \cdot (1 - \rho _{s_1, s_2}) \\&\rho _{s_1, s_2} + \rho _{s_2, s_1} = 1 \end{aligned} \end{aligned}$$

#### Assignment-time constraints

- *The arrival and departure times of the truck from the gate is 0 if they are not assigned to each other.*
  For each truck *v* and gate *g*:
  6 $$\begin{aligned} ga_{v, g} \le gd_{v, g} \le M_v \cdot x_{v, g} \end{aligned}$$
- *Lower and upper bounds on arrival and departure times of the trucks.*
  For each truck *v*:
  1. Arrival at parking lot cannot be earlier than planned.
    7 $$\begin{aligned} pV(v) \le pa_{v} \end{aligned}$$
  2. The arrival in the parking lot must precede the departure from the parking lot.
    8 $$\begin{aligned} pa_{v} \le pd_{v} \end{aligned}$$
  3. The departure from the parking lot must precede the arrival at the gate.
    9 $$\begin{aligned} pd_{v} \le \sum _g ga_{v, g} - \sum _g tG(g) \cdot x_{v,g} \end{aligned}$$
  4. The arrival at the gate must precede the departure from the gate.
    10 $$\begin{aligned} \sum _g ga_{v, g} \le \sum _g gd_{v, g} - \sum _g tVG(v, g) \cdot x_{v,g} \end{aligned}$$
  5. The departure from the gate must precede the arrival at the dock.
    11 $$\begin{aligned} \sum _g gd_{v, g} \le da_{v} - \sum _g tGD(g, aVD(v)) \cdot x_{v,g} \end{aligned}$$
  6. Arrival at the dock must precede departure from the dock.
    12 $$\begin{aligned} da_{v} \le dd_{v} - tVD(v) \end{aligned}$$
  7. The departure from the dock must occur before the latest possible departure time.
    13 $$\begin{aligned} dd_{v} \le eV(v) \end{aligned}$$
- *The container can be placed onto its associated ship before the ship leaves the dock.*
  Past the departure of the truck *v* from the dock ($dd_{v}$), truck *v* is considered to be removed from the system, and only a container remains. In this article, container loading is simplified and only parameter *tVS*(*v*) is considered that represents the amount of time required to load the truck container *v* onto the ship. It is assumed that containers can be loaded in parallel and there is no capacity limit on the number of simultaneous containers loading onto the ship.
  We define a constraint that ensures that every container can be placed onto its associated ship before the ship leaves the dock. For each truck *v*:
  14 $$\begin{aligned} \sum _s aVS(v, s) \cdot (dd_{v} + tVS(v)) \le \sum _s aVS(v, s) \cdot sd_{s} \end{aligned}$$
  (*Another variant*) For each truck *v* and ship *s* such that $aVS(v, s) = 1$:
  15 $$\begin{aligned} dd_{v} \le sd_{s} - tVS(v) \end{aligned}$$
- *Lower and upper bounds on ship arrival and departure times:*
  For each ship *s*:
  16 $$\begin{aligned} pS(s) \le sa_{s} \le sd_{s} \le eS(s) \end{aligned}$$

#### Capacity constraints

- *Auxiliary variable:*
  For this Sect. 3.3.4 and the following Sect. 3.3.5 we introduce a new auxiliary binary variable $\upsilon _{v, w, g}$ that represents the assignment of a pair of trucks *v* and *w* and a gate *g*. That is, for each pair of trucks $v, w \in V$ such that $v \ne w$ and gate $g \in G$
  $$\begin{aligned} \upsilon _{v, w, g} = x_{v, g} \cdot x_{w, g}, \end{aligned}$$
  which can be defined in the following way:
  17 $$\begin{aligned} \begin{aligned}&\upsilon _{v, w, g} \le x_{v, g} \\&\upsilon _{v, w, g} \le x_{w, g} \\&\upsilon _{v, w, g} \ge x_{v, g} + x_{w, g} - 1 \end{aligned} \end{aligned}$$
- *Parking lot:*
  For each pair of trucks $v, w \in V$ such that $v \ne w$, we define auxiliary binary variables $\alpha _{v, w}$, $\beta _{v, w}$, and $\gamma _{v, w}$. The variable $\gamma _{v, w} = 1$ if:
  $$\begin{aligned} pa_{w} \le pa_{v} \le pd_{w}, \end{aligned}$$
  indicating that the arrival time of truck *v* falls within the parking time interval of truck *w*. If any equality holds (i.e., $pa_{w} = pa_{v}$ or $pa_{v} = pd_{w}$), we enforce a lexicographic order, that is $\gamma _{v, w} = 1$ and $\gamma _{w, v} = 0$ if $v < w$. Let $\gamma _{v, w} = 0$ otherwise.
  The other two variables, $\alpha _{v, w}$ and $\beta _{v, w}$, represent the precedence relations for each part of the aforementioned inequality such that $\gamma _{v, w} = \alpha _{v, w} \cdot \beta _{v, w}$:
  - $\alpha _{v, w} = 1$ if $pa_{w} \le pa_{v}$ and $\alpha _{v, w} = 0$ otherwise, and in the case $pa_{w} = pa_{v}$ let $\alpha _{v, w} = 1$ and $\alpha _{w, v} = 0$ if $v < w$ in a lexicographic order.
  - $\beta _{v, w} = 1$ if $pa_{v} \le pd_{w}$ and $\beta _{v, w} = 0$ otherwise, and in the case $pa_{v} = pd_{w}$ let $\beta _{v, w} = 1$ and $\beta _{w, v} = 0$ if $v < w$ in a lexicographic order.
  The above are guaranteed by the following constraints. For each pair of trucks $v, w \in V$ such that $v \ne w$:
  18 $$\begin{aligned} \begin{aligned}&pa_{w} - pa_{v} \le M_v \cdot (1 - \alpha _{v, w}) \\&pa_{v} - pd_{w} \le M_v \cdot (1 - \beta _{v, w}) \\&\alpha _{v, w} + \alpha _{w, v} = 1 \end{aligned} \end{aligned}$$
  And the linearization of $\gamma _{v, w} = \alpha _{v, w} \cdot \beta _{v, w}$, together with the constraint for precedence, can be achieved in the following way:
  19 $$\begin{aligned} \begin{aligned} \gamma _{v, w}&\le \alpha _{v, w} \\ \gamma _{v, w}&\le \beta _{v, w} \\ \gamma _{v, w}&\ge \alpha _{v, w} + \beta _{v, w} - 1 \\ \gamma _{v, w}&+ \gamma _{w, v} \le 1 \end{aligned} \end{aligned}$$
  Then the capacity constraint can be expressed as follows. For each truck $v \in V$ we ensure that a truck can enter the parking lot only if there are currently less than $cP - 1$ trucks in the parking lot:
  20 $$\begin{aligned} \sum _{w \in V, w \ne v} \gamma _{v, w} \le cP - 1 \end{aligned}$$
  The above set of constraints (18–20) is enough to ensure the capacity limit for the parking lot. Consider any subset of trucks $W \subseteq V$ that have a common non-empty intersection of intervals $I = \bigcap _{v \in W} [pa_v, pd_v]$, $I \ne \emptyset$. Choose $V'$ out of all such *W*’s with the most amount of trucks. By construction, there are at least two trucks $v, w \in V'$ such that $I = [pa_v, pd_w]$. In case there are several trucks like *v*, that are $v_1, v_2, \ldots , v_k \in V'$ such that
  $$\begin{aligned} pa_v = pa_{v_1} = pa_{v_2} = \ldots = pa_{v_k}, \end{aligned}$$
  truck *v* is chosen to be the smallest in a lexicographic order out of all such trucks $v_1, v_2 \ldots , v_k$.
  In this case, by definition, $\alpha _{v, u} = \beta _{v, u} = 1$ for a chosen truck *v* and any $u \in V'$, $u \ne v$. Thus, $\gamma _{v, u} = \alpha _{v, u} \cdot \beta _{v, u} = 1$ for any $u \in V'$, $u \ne v$, implying that limiting the capacity of the parking lot, which is by construction, limiting the size of the subset of intersecting trucks $V'$, is equivalent to limiting the sum over all $\gamma$’s for the chosen truck *v*:
  $$\begin{aligned} \sum _{u \in V', u \ne v} \gamma _{v, u} \le cP - 1 \end{aligned}$$
  Since for any truck $u \in V\setminus V'$, $\gamma _{v, u} = 0$ by definition, there are no unnecessary constraints put on other trucks outside of the intersecting ones. Thus,
  $$\begin{aligned} \sum _{u \in V', u \ne v} \gamma _{v, u} = \sum _{u \in V, u \ne v} \gamma _{v, u} \le cP - 1. \end{aligned}$$
  Of course, since $pa_v$ and $pd_v$ are variables for any $v \in V$, the size of $V'$ is not known in advance, but enforcing the above inequality for every intersecting subset of trucks *W* is equivalent to considering the aforementioned inequality only for the maximum, in terms of the number of trucks, subset $V'$.
  Considering the above inequality over all intersecting subsets *W* is equivalent to considering it for all trucks $v \in V$, which precisely gives us inequality (20). This concludes the proof that the considered set of constraints is enough to ensure the capacity limit for the parking lot. Similarly, capacity constraints are constructed for the roads (to the gates (21–24) and to the docks (25–28)).
- *Roads from parking lot to gates:*
  For each pair of trucks $v, w \in V$ such that $v \ne w$:
  21 $$\begin{aligned} \begin{aligned}&pd_{w} - pd_{v} \le M_v \cdot (1 - \delta _{v, w}) \\&pd_{v} - \sum _{g \in G} ga_{w, g} \le M_v \cdot (1 - \theta _{v, w}) \\&\delta _{v, w} + \delta _{w, v} = 1 \end{aligned} \end{aligned}$$
  The equation $\eta _{v, w} = \delta _{v, w} \cdot \theta _{v, w}$ can be linearized in the following way. Together with the constraint for precedence, for each pair of trucks $v, w \in V$ such that $v \ne w$ we get:
  22 $$\begin{aligned} \begin{aligned} \eta _{v, w}&\le \delta _{v, w} \\ \eta _{v, w}&\le \theta _{v, w} \\ \eta _{v, w}&\le \delta _{v, w} + \theta _{v, w} - 1 \\ \eta _{v, w}&+ \eta _{w, v} \le 1 \\ \end{aligned} \end{aligned}$$
  Then for each gate $g \in G$ and truck $v \in V$:
  23 $$\begin{aligned} \sum _{w \in V, w \ne v} \zeta _{v, w, g} \le cG(g) - 1, \end{aligned}$$
  such that $\zeta _{v, w, g}$ is a binary variable equal to 1 if $\eta _{v, w} = 1$ for trucks *v* and *w* that are going to the same gate, that is all three variables $\eta _{v, w}$, $x_{v, g}$, and $x_{w, g}$ are 1, and $\zeta _{v, w, g} = 0$ otherwise. This can be represented in the following way. For each gate $g \in G$, truck $v \in V$ and $w \in W$:
  $$\begin{aligned} \begin{aligned} \zeta _{v, w, g}&= \eta _{v, w} \cdot x_{v, g} \cdot x_{w, g} \\&= \eta _{v, w} \cdot \upsilon _{v, w, g}, \end{aligned} \end{aligned}$$
  which can be then linearized as follows. For each gate $g \in G$, truck $v \in V$ and $w \in W$:
  24 $$\begin{aligned} \begin{aligned}&\zeta _{v, w, g} \le \eta _{v, w} \\&\zeta _{v, w, g} \le \upsilon _{v, w, g} \\&\zeta _{v, w, g} \ge \eta _{v, w} + \upsilon _{v, w, g} - 1 \end{aligned} \end{aligned}$$
- *Roads between gates and docks:*
  For each pair of trucks $v, w \in V$ such that $v \ne w$ and $aVD(v) = aVD(w)$:
  25 $$\begin{aligned} \begin{aligned}&\sum _{g \in G} gd_{w, g} - \sum _{g \in G} gd_{v, g} \le M_v \cdot (1 - \mu _{v, w}) \\&\sum _{g \in G} gd_{v, g} - da_{w} \le M_v \cdot (1 - \nu _{v, w}) \\&\mu _{v, w} + \mu _{w, v} = 1 \\&\nu _{v, w} + \nu _{w, v} = 1 \end{aligned} \end{aligned}$$
  The equation $\xi _{v, w} = \mu _{v, w} \cdot \nu _{v, w}$ can be linearized in the following way. Together with the constraint for precedence, for each pair of trucks $v, w \in V$ such that $v \ne w$ and $aVD(v) = aVD(w)$:
  26 $$\begin{aligned} \begin{aligned} \xi _{v, w}&\le \mu _{v, w} \\ \xi _{v, w}&\le \nu _{v, w} \\ \xi _{v, w}&\le \mu _{v, w} + \nu _{v, w} - 1 \\ \xi _{v, w}&+ \xi _{w, v} \le 1 \end{aligned} \end{aligned}$$
  Then for each gate $g \in G$, truck $v \in V$:
  27 $$\begin{aligned} \sum _{w \in V, w \ne v, aVD(v) = aVD(w)} \chi _{v, w, g} \le (cGD(g, aVD(v)) - 1), \end{aligned}$$
  such that $\chi _{v, w, g}$ is a binary variable that is equal to 1 if all three variables $\xi _{v, w}$, $x_{v, g}$, and $x_{w, g}$ are 1 and $\chi _{v, w, g} = 0$ otherwise. That is
  $$\begin{aligned} \begin{aligned} \chi _{v, w, g}&= \xi _{v, w} \cdot x_{v, g} \cdot x_{w, g} \\&= \xi _{v, w} \cdot \upsilon _{v, w, g}, \end{aligned} \end{aligned}$$
  which can be linearized in the following way. For each gate $g \in G$, for each pair of trucks $v, w \in V$ such that $v \ne w$ and $aVD(v) = aVD(w)$:
  28 $$\begin{aligned} \begin{aligned}&\chi _{v, w, g} \le \xi _{v, w} \\&\chi _{v, w, g} \le \upsilon _{v, w, g} \\&\chi _{v, w, g} \ge \xi _{v, w} + \upsilon _{v, w, g} - 1 \end{aligned} \end{aligned}$$

#### Precedence constraints

- *Roads from parking lot to gates:*
  For each pair of trucks $v, w \in V$ such that $v \ne w$ and we define a binary variable $\phi _{v, w} = 1$ for trucks that go to the same gate if
  $$\begin{aligned} pd_{v} \le pd_{w}, \end{aligned}$$
  that is, if the truck *v* departs from the parking lot before the truck *w*. If $pd_{v} = pd_{w}$, we enforce lexicographic order, that is $\phi _{v, w} = 1$ and $\phi _{w, v} = 0$ if $v < w$, and we set $\phi _{v, w} = 0$ otherwise.
  To ensure that trucks *v* and *w* maintain relative order, we should consider their relative position before they enter gate *g* and compare it with previously defined variable $\phi _{v, w}$.
  For each pair of trucks $v, w \in V$ such that $v \ne w$ and gate $g \in G$ we introduce two auxiliary variables:
  $$\begin{aligned} \omega _{v, w, g}&= (pd_{v, g} - pd_{w, g}) \cdot x_{v, g} \cdot x_{w, g} \\ &= (pd_{v, g} - pd_{w, g}) \cdot \upsilon _{v, w, g}\\ \tau _{v, w, g}&= (ga_{v, g} - ga_{w, g}) \cdot x_{v, g} \cdot x_{w, g} \\ &= (ga_{v, g} - ga_{w, g}) \cdot \upsilon _{v, w, g}, \end{aligned}$$
  which can be linearized in the following way:
  29 $$\begin{aligned} \omega _{v, w, g} &\le \upsilon _{v, w, g} \cdot M_v \nonumber \\ \omega _{v, w, g} &\ge - \upsilon _{v, w, g} \cdot M_v \nonumber \\ \omega _{v, w, g} &\le (pd_{v, g} - pd_{w, g}) + (1 - \upsilon _{v, w, g}) \cdot M_v \nonumber \\ \omega _{v, w, g} &\ge (pd_{v, g} - pd_{w, g}) - (1 - \upsilon _{v, w, g}) \cdot M_v \end{aligned}$$
  30 $$\begin{aligned} \tau _{v, w, g} &\le \upsilon _{v, w, g} \cdot M_v \nonumber \\ \tau _{v, w, g} &\ge - \upsilon _{v, w, g} \cdot M_v \nonumber \\ \tau _{v, w, g} &\le (ga_{v, g} - ga_{w, g}) + (1 - \upsilon _{v, w, g}) \cdot M_v \nonumber \\ \tau _{v, w, g} &\ge (ga_{v, g} - ga_{w, g}) - (1 - \upsilon _{v, w, g}) \cdot M_v \end{aligned}$$
  Then we can define such a precedence relation in the following way. For each pair of trucks $v, w \in V$ such that $v \ne w$:
  31 $$\begin{aligned} \begin{aligned} \sum _{g \in G} \omega _{v, w, g}&\le M_v \cdot (1 - \phi _{v, w}) \\ \sum _{g \in G} \tau _{v, w, g}&\le M_v \cdot (1 - \phi _{v, w}) \\ \phi _{v, w}&+ \phi _{w, v} = 1 \end{aligned} \end{aligned}$$
- *Roads between gates to docks:*
  In this case, we can use the fact that for each truck and their respective gate, the departure time from this gate is the sum of gate arrival and gate processing time. Since each gate can process only one truck at a time, it means that if a pair of trucks arrive at the same gate in a certain order, we know that they depart in the same order as well. That is why we can use a previously defined variable $\phi _{v, w}$ that represents such a relative order before entering the gate and compare it with the order of trucks before entering the dock.
  This can be achieved in the following way. For each pair of trucks $v, w \in V$ such that $v \ne w$ and $aVD(v) = aVD(w)$:
  32 $$\begin{aligned} \begin{aligned}&da_{v} - da_{w} \le M_v \cdot (1 - \phi _{v, w}) \\ \end{aligned} \end{aligned}$$

### Objective function

Minimization of costs resulting from the cumulative operation time of trucks:

33 $$\begin{aligned} \begin{aligned} \text {Minimize}&\sum _{v \in V} uV(v) \cdot \left( dd_{v} - pV(v) \right) \end{aligned} \end{aligned}$$

## Rolling horizon heuristic

In this work, we implemented a rolling horizon to manage the complexity of the problem. Initially, a set of trucks $V$ is given, which are sorted by their planned arrival time $pV(v)$, for each $v \in V$. This sorted array is split into smaller arrays, sorted by arrival time, which are called horizons. The problem is then solved sequentially for each horizon, using the solution from the previous horizon to inform the next. Notice that horizons are not necessarily independent, that is, depending on the parameter *w* (which will be introduced later) two consecutive horizons might intersect.

Formally, let $H$ be the sequence of time horizons $H_i \subseteq V$ for any *i* ordered by arrival, so

$$\begin{aligned} H = (H_1, H_2, \ldots , H_n), \end{aligned}$$

and for any $i, j \in \{ 1, 2, \ldots , n \}$, $i < j$:

$$\begin{aligned} \max _{v \in H_i \setminus H_j} pV(v) \le \min _{v \in H_j \setminus H_i} pV(v). \end{aligned}$$

**Fig. 2:**
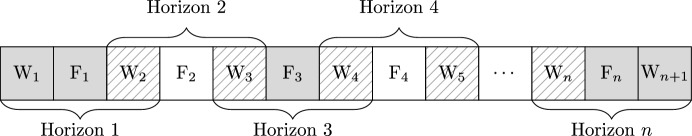
Illustration of the rolling horizon heuristic

For each $i \in \{ 1, 2, \ldots , n \}$ we split horizon $H_i$ into 3 distinct subsets in the following way:

$$\begin{aligned} H_i = W_i \cup F_i \cup W_{i + 1}, \end{aligned}$$

such that

$$\begin{aligned} W_{i + 1} = H_i \cap H_{i + 1}, \end{aligned}$$

for $i \in \{ 1, 2, \ldots , n - 1 \}$. Using this approach, a sequence of intersecting horizons is generated that allows us to control the size of their intersection (see Fig. 2).

We assign to each interval $F_i$ and $W_j$ a number of trucks such that the size of each interval is influenced by a predefined intersection percentage $w> 0$, such that $|W_i|$ is approximately equal to $w \cdot |F_i|$ for any *i*. By construction, number of trucks per horizon and for each $F_i$ and $W_j$ are natural numbers, while *w* might be not. Moreover, the total number of trucks might not necessarily be divisible by the number of intervals considered for the RH heuristic. Because of that, we propose the following approach that provides us with horizons of approximately equal sizes, which translates into sub-problems of roughly equal complexity, while still maintaining the control over the number of trucks in the intersection between the horizons. Given $V$, the set of trucks, and $n$, the number of horizons, the sizes of $F_i$ and $W_i$ are computed as follows:

1. Let $n_{\text {total}} = n + (n + 1) \cdot w$, be the total, adjusted by *w*, count of partitions.
2. Let $t_{\text {base}} = \left\lfloor \frac{V}{n_{\text {total}}} \right\rfloor$, be the base number of trucks per partition.
3. Let $t_{\text {remainder}} = V - t_{\text {base}} \cdot n_{\text {total}}$, be the remainder of trucks after the initial distribution.
4. Each $F$ interval size is determined as:
  $$\begin{aligned} |F| = t_{\text {base}} + \left\lfloor \frac{t_{\text {remainder}}}{n} \right\rfloor \end{aligned}$$
5. Each $W$ interval size is determined as:
  $$\begin{aligned} |W| = \left\lfloor t_{\text {base}} \cdot w \right\rfloor \end{aligned}$$
6. The final adjustment is made by distributing any remaining trucks ($t_{\text {remainder}} \mod n$) to the last $W$ interval ($W_{n + 1}$), ensuring all trucks are included.

To minimize the number of potential conflicts between horizons, which could lead to an infeasible overall solution, intersecting horizons are chosen instead of independent ones (that is $W_i = \emptyset$ for every *i*). We utilize the intersecting regions, $W_i$, in the following way:

1. Solve the problem for the initial horizon $H_1 = [ W_1, F_1, W_2 ]$ and extract the partial solution $S_1$ for the problem restricted to the first horizon $H_1$ to guide the subsequent stages of the process.
2. For $i = 2, 3, \ldots , n$ repeat the following:
  - Solve the problem over the horizon $[ H_{i - 1}, H_i ]$ with a set of fixed variables $[ W_{i - 1}, F_{i - 1} ]$ (provided as additional constraints with fixed values from the previously obtained solution $S_{i - 1}$) and a set of variables $W_i$ to warm start the model and allow them to be modified.
  - Obtain a solution $S_i$ and merge it with a previously obtained solution $S_{i - 1}$, replacing the solution values of the trucks’ variables in the interval $W_i$.
  - (Optional) As a recommendation, keep track of the global variables, e.g., total runtime, which would be a sum of runtimes for all the horizons.

An important parameter to consider is the intersection percentage. Increasing the relative size of the warm start part gives flexible horizons in which trucks could be modified several times (except the first and last horizons), which leads to more robust interval solutions based on our experience. Having large enough fixed parts of the horizons helps to find an initial feasible solution for the subsequent interval quicker and based on our experience leads to better performance. However, leaving fixed part or warm part too large can have a negative impact due to either too strict constraints or a much larger problem to solve. In our case, we choose the intersection percentage *w* to be 1, thus having a balanced configuration where approximately half of the trucks of intermediate horizons (that are not the first and last horizons) belong to the warm start parts and the rest belongs to the fixed parts.

## Results

In this section, we evaluate our model on simulated instances and demonstrate the results. The input instances were prepared in a semi-randomized way, mixing real-world data based on the port of Koper (Slovenia) and simulated truck instances.

### Input instances

The port structure was fixed for all the tests and is based on the structure of the port of Koper, featuring 3 gates and 4 docks, with each gate connected to every dock. Other parameters, such as road capacities, parking lot size, road lengths, and processing times at gates and docks, are based on semi-random data.

To evaluate the model’s performance, truck arrivals were simulated using a specific probability distribution. The beta distribution $\text {beta}(1.29, 3.32, [0, 10])$ (Yang et al. 2010) was chosen to accurately represent the arrival times of trucks during peak periods and to model the uneven nature of these events, which are common in ports where most trucks arrive at the beginning of narrow time windows. Unlike the traditional four-step demand model (Yang et al. 2007), this distribution addresses the fluctuations of traffic flow within a port, as highlighted by Yang et al. (2010). For each truck, the departure time is set at 5 hours after its arrival. The objective for the trucks is to minimize their time in the port, so delays of trucks are already considered. This type of departure time serves just as a generous deadline and is one of the input parameters, thus can be chosen in order to satisfy the practical needs of the specific port environment.

External factors such as bad weather conditions, urban traffic congestion, and changes in work shifts also significantly influence truck arrivals (Yang et al. 2010), although they are not covered in this simulation.

For ship arrivals and departures, real-world data was used from MarineTraffic Live Ships Map (2024) for the port of Koper. Based on the data from Statistical Office of the Republic of Slovenia (SURS) (Statistical Office 2022), 1183 ships for the purpose of container transportation arrived in the port of Koper in the year 2022. This gives us approximately 3 to 4 ship arrivals per day. Our tests cover sets of 3, 4, and 5 ships that arrived at the port of Koper consecutively over the span of 1 to 2 days.

Each configuration is defined as a tuple (number of ships, number of trucks per ship). For each configuration, we generated 5 different sets of truck data (referred to as *repetitions*), which are different from each other due to the inherent randomness in truck generation based on beta distribution and assigning unit costs to trucks. The results, such as average runtime and average objective value, presented in Sect. 5.2, are computed by taking a mean value across all repetitions. Specifically, for each repetition, we generated truck arrivals using different random seeds from the beta distribution. For each truck, a random integer was uniformly selected from a closed interval, representing the unit cost of using the truck (parameter *uV*(*v*) for $v \in V$).

Our objective is to evaluate the efficiency of the model across various scenarios, starting from simple but not trivial ones with small amounts of trucks, until the computer capabilities of finding at least one feasible solution in a reasonable time.

Based on our experiments, we determined the limits on the complexity of the problem to reach optimal solution using the basic model without any additional heuristics (that is about 200 trucks in total), and we introduce the following configurations: numbers of ships (ranging from 3 to 5), trucks per ship (ranging from 10 to 50 with increments of 10), and number of time intervals (1, 5, and 10) from the *rolling horizon* heuristic (outlined in Sect. 4). The scenario with only 1 time interval serves as a baseline, representing the basic model without any heuristics used.

With this approach, we can observe the growth in complexity of the problem and how it is related to the parameters of the solutions, such as runtime, MIP optimality gap and more. We also compare objective values between optimal and heuristic solutions in order to analyze rolling horizon performance. To better understand the practicality of the rolling horizon technique, we keep track of the instances that were not able to find any feasible solution under the given time limit or if the model incorrectly arrived at the infeasible solution. The results will be presented in the form of three tables, each summarizing different types of data, with the discussion of edge cases in the following section 5.2.

### Computational results

We conducted computational experiments using the Gurobi 10.0.3 solver on an AMD Ryzen 7 5800*H* 3.2 GHz CPU and a 16 GB RAM setup. The time limit for the tests was set to 60 minutes.

Table 1 focuses on checking the computational boundaries for the base model (a single interval). Each input configuration is defined as a 2-tuple (number of ships, number of trucks per ship), and the number of optimal (OPT) and suboptimal (SS) solutions is given for them, as well as the number of instances where no feasible solution was achieved within the time limit (no FUT). The table also presents the average gap of SS and the overall average runtime of FUT.

**Table 1:** Aggregated results of the base model for different configurations

Instance		Metrics				
Ships	Trucks per ship	Optimal solution (OPT)	Suboptimal solution (SS)	No feasible solution in time limit (no FUT)	Avg gap of SS (%)	Avg runtime of FUT (s)
3	10	5	0	0	NaN	1.96
	20	5	0	0	NaN	43.89
	30	5	0	0	NaN	305.79
	40	4	1	0	0.21	1683.70
	50	1	4	0	0.98	3475.85
4	10	5	0	0	NaN	5.71
	20	5	0	0	NaN	95.68
	30	5	0	0	NaN	1254.67
	40	4	1	0	0.57	2812.69
	50	0	5	0	0.98	3610.97
5	10	5	0	0	NaN	8.31
	20	5	0	0	NaN	246.32
	30	5	0	0	NaN	1785.64
	40	1	4	0	0.71	3582.92
	50	0	1	4	1.11	3601.87

Table 2 presents the results of the RH heuristic with 5 and 10 intervals. For each configuration, the table gives the number of instances where the heuristic found the optimal solution of the base model (found OPT), the cases where it gave a better result than the suboptimal solution of the base model (better than SS), and the cases where no feasible solution was found under the time limit (no FUT). Average running times are also given for feasible solutions. The heuristic never performed worse than the base model; it either found the optimal solution or provided better ones than the SS of the base model.

**Table 2:** Aggregated results of the RH heuristic for different configurations with 5 and 10 intervals

Instance			Metrics			
Ships	Trucks per ship	Intervals	Found OPT	Better than SS	No feasible solution in time limit (no FUT)	Avg runtime of FUT (s)
3	10	5	5	0	0	0.99
		10	5	0	0	0.75
	20	5	5	0	0	7.55
		10	5	0	0	3.42
	30	5	5	0	0	28.16
		10	5	0	0	9.97
	40	5	4	1	0	92.74
		10	2	1	2	29.46
	50	5	1	3	1	237.68
		10	1	4	0	91.77
4	10	5	5	0	0	1.88
		10	5	0	0	1.06
	20	5	5	0	0	14.89
		10	5	0	0	5.74
	30	5	4	1	0	67.68
		10	5	0	0	23.59
	40	5	4	1	0	220.33
		10	4	1	0	57.56
	50	5	0	5	0	440.47
		10	0	5	0	139.71
5	10	5	5	0	0	3.88
		10	5	0	0	1.67
	20	5	5	0	0	30.02
		10	5	0	0	10.41
	30	5	4	0	1	109.78
		10	4	1	0	43.56
	40	5	1	4	0	364.25
		10	1	4	0	104.69
	50	5	0	3	2	1137.50
		10	0	5	0	320.94

It can be seen from Tables 1 and 2 that for smaller configurations (under 30 trucks per ship), optimal solutions were achieved with at least one of the interval settings for all repetitions. Acquiring an optimal solution for the larger instances is more difficult (e.g. configurations (3, 50), (5, 40)), or sometimes even impossible (e.g. configurations (4, 50), (5, 50)). Although feasible (but suboptimal) solutions were found for almost all such instances, case (5, 50) shows the computational limits of the base model. In such cases, it would be beneficial, or even necessary, to use heuristics. While feasible solutions can be achieved for all repetitions of (5, 50) with the use of 10 intervals in the RH heuristic, the base model was able to find a feasible solution for only 1 out of 5 repetitions. It can happen that some instances are solved and proved to be infeasible, but among all of the instances here we did not encounter such a case.

The gap of suboptimal solutions for the base model is always less than $2.5\%$ and, on average, is about $1\%$. For the configurations that were solved to optimality with the base model, it can be noticed that most of the heuristic solutions were able to find optimal solutions as well. In some cases where the base model was able to find a feasible solution, some RH solutions were infeasible, but in all such cases at least one of the RH configurations (either 5- or 10-interval) was able to find a feasible solution. This implies that using heuristic solutions in cases where an optimal solution is not achievable might be a good overall approximation strategy. In the cases where the base model only found an SS solution, the RH solution was always better than this SS.

We can notice a huge increase in runtime performance with increasing the number of intervals. Given the approximation gap mentioned above, it is recommended to solve an instance with the RH heuristic at first (using different numbers of intervals or different intersection percentages) until obtaining a feasible solution. If needed, it is then possible to warm-start the base model using the feasible interval solution. The worst case, that is, the warm-started model not being able to use a starting feasible interval solution, would be equivalent to just running the base model by itself.

#### Analysis of instance structure and complexity

It can be noticed that the base model performed significantly worse for configuration (3, 50) than for configuration (5, 30), while having the same total number of trucks. This leads us to believe that the complexity of the instance depends not only on the total number of vehicles, but also on the specific truck distribution. Since truck arrivals for each ship follow beta distributions, overlapping peaks from multiple distributions can create noticeable spikes in truck arrival numbers during certain time intervals. This results in a higher demand for limited port resources within short time periods, which increases the complexity of the instance.

**Fig. 3:**
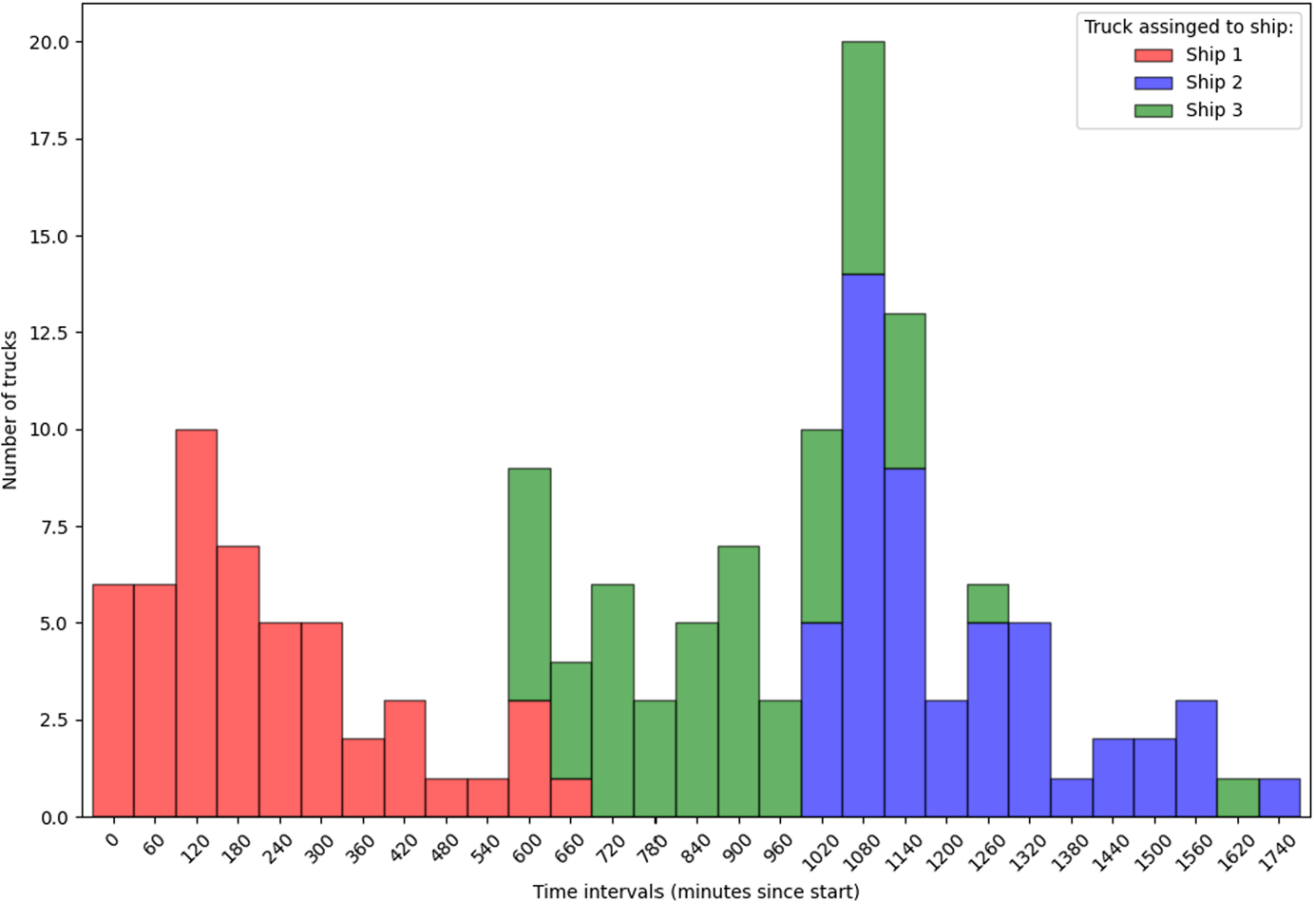
Stacked histogram of number of truck arrivals per 60-minute interval. Configuration: (3 ships, 50 trucks per ship), repetition 3

**Fig. 4:**
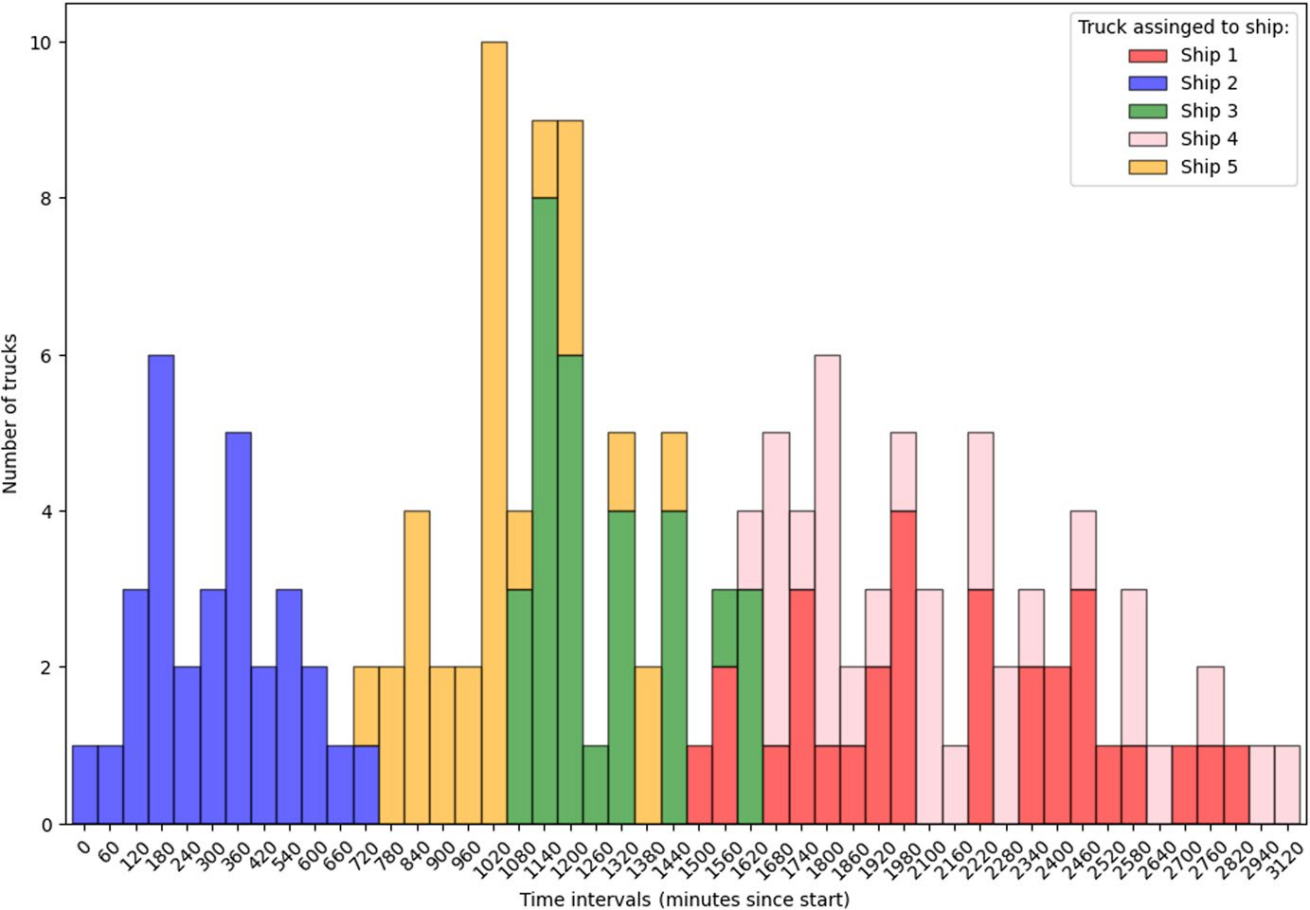
Stacked histogram of number of truck arrivals per 60-minute interval. Configuration: (5 ships, 30 trucks per ship), repetition 3

For example, consider Fig. 3 that shows the distribution of truck arrivals, grouped into 60-minute time intervals over the entire planning horizon for configuration (3, 50). We choose repetition 3 as it has the highest MIP gap (of $2.2\%$) among all other repetitions. We can compare it with the distribution for the (5, 30) configuration (see Fig. 4), which, despite the noticeable spikes, has less pronounced peaks in terms of the number of trucks and was solved optimally with the base model.

#### Analysis of rolling horizon performance

While the overall results of the RH heuristic were presented in Table 2, this subsection focuses on comparing the use of different number of intervals for the rolling horizon heuristics (see Table 3). We analyzed various configurations of RH using different number of trucks per interval and different values of parameter *w*. After experimenting with various interval numbers, we ended up using 5 and 10, as those seemed to provide the best representation for our inputs. However, other division numbers also worked with comparable results. Similarly, as mentioned in Sect. 4, we decided to choose a balanced approach of $w = 1$ based on our experience.

In Table 3, we demonstrate the trade-off between potential increase in objective costs versus the reduction in running time. That is, for each configuration we can observe the number of instances where 10-interval solutions are worse than 5-interval solutions and how much they are worse in such cases. As mentioned in the previous table, the difference in objectives with the base model is never more than 0, and comparing interval solutions costs revealed that they are identical except 1 instance. In this instance (case (5, 30)), the difference is marginal, that is ${3.94} \times 10^{-4}$, which leads us to believe that the feasible RH solutions are most likely optimal, but within the solver tolerance.

**Table 3:** Trade off between potential increase in costs versus reduction in running time

Configuration		Metrics				
Ships	Trucks per ship	10 worse than 5	Avg extra cost (%)	Avg runtime reduction (%)	5 only cases	10 only cases
3	10	0	NaN	24.52	0	0
	20	0	NaN	55.37	0	0
	30	0	NaN	63.86	0	0
	40	0	NaN	40.70	2	0
	50	0	NaN	53.58	0	1
4	10	0	NaN	43.59	0	0
	20	0	NaN	61.29	0	0
	30	0	NaN	64.25	0	0
	40	0	NaN	72.83	0	0
	50	0	NaN	68.17	0	0
5	10	0	NaN	57.10	0	0
	20	0	NaN	64.90	0	0
	30	1	3.94e$-$04	53.44	0	1
	40	0	NaN	71.33	0	0
	50	0	NaN	46.38	0	2

**Fig. 5:**
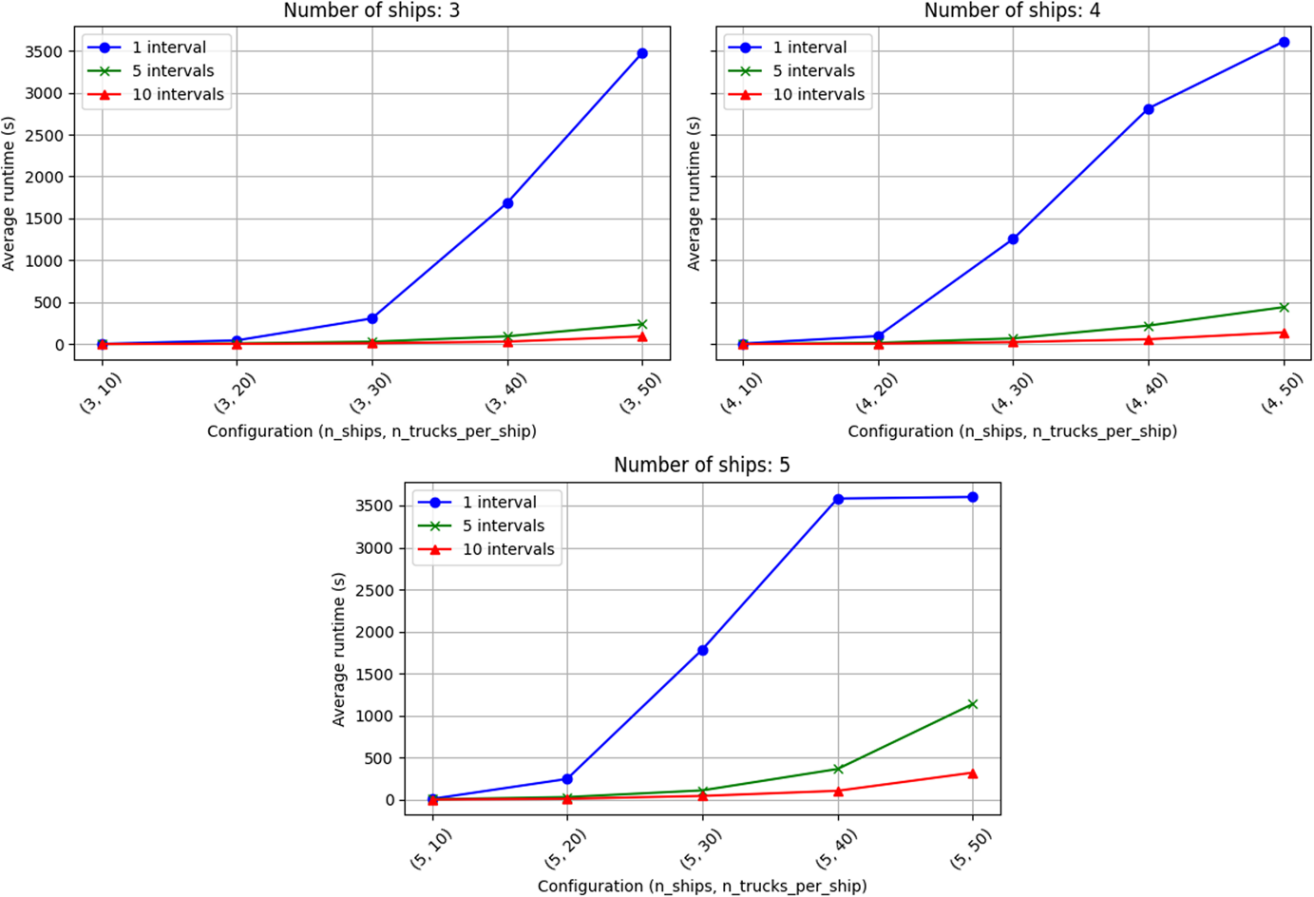
Average runtime by configuration (a tuple (number of ships, number of trucks per ship)) and number of intervals

Although the extra costs are marginal, the reduction in time can be very noticeable (approximately $55\%$ on average). The runtime results could be better observed in the form of a line chart (Fig. 5).

Also, based on the last two columns of Table 3 it can be noticed that there are cases in which either 5-interval or 10-interval found a feasible solution, while the other did not. Because of this, we recommend starting with a higher number of intervals first (from experience, 15–20 trucks per interval can be a good starting option) and go in decreasing order over the number of intervals until the feasible solution is found.

#### Managerial implications

While TAS offer an existing way for ports to schedule their truck arrivals (Caballini et al. 2020), we do not intend our model to replace these current existing practices. We intentionally designed the model to be general and flexible and to solve a more detailed scheduling problem than the assignment given by a TAS. Moreover, the solution that our approach provides can be used as a decision support tool by the experts at the port to make substantiated choices and check the efficiency of schedules given by the TAS purely from the port’s perspective. There are several managerial implications similar to TAS’s, such as incentivizing high-volume drayage firms, understanding how terminal resource utilization and customer time-windows affect drayage costs, and practicality of the information sharing policies between container terminal operators and drayage firms (Torkjazi et al. 2018). Concerning the information privacy, our model is intended to be applied by port operators only using limited information from drayage firms, that is about expected truck arrivals. Considering other implications, our model can provide a feasible overall solution (optimal or close to optimal), which can then be used as a practical reference point for TAS implementations, while also keeping the complexity manageable for real-world applications.

## Conclusion

This paper addressed the problem of optimizing container flow in port logistics, focusing on the truck scheduling aspect of the port environment. We developed a mixed-integer linear programming (MILP) model to streamline port processes, including truck arrival, movement within the port, and container unloading. Simulations based on realistic scenarios demonstrated the model’s ability to provide optimal solutions for simulated instances with about 200 trucks and close to optimal solutions for larger instances using rolling-horizon heuristic within the time limit of 60 minutes.

For the medium size port of Koper (Slovenia), based on data by Krmac and Djordjević (2023), it is expected to have approximately 83 trucks per ship. Even though we are not able to determine the optimum with the base model of the typical ”one day problem” for this real-world scenario ((3, 80) configuration), we used rolling horizon with 15 and 10 interval configurations and were able to find feasible solutions for all of these instances. So, our further research could investigate the model’s performance over longer planning horizons and incorporate other metaheuristic algorithms for solving larger, more complex instances.

Furthermore, validation of the model with real-world truck arrival data would be valuable in evaluating its practical applicability. We should also continue our extensive testing of the current model for the different port structures, truck-ship configurations, various values of the intersection percentage parameter of the rolling-horizon heuristic, among others. All of that would benefit our understanding of computational limits of the model.

## References

[CR1] Abdelmagid AM, Gheith M, Eltawil A (2022) Scheduling external trucks appointments in container terminals to minimize cost and truck turnaround times. Logistics 6(3):45

[CR2] Abdelmagid AM, Gheith MS, Eltawil AB (2022) A comprehensive review of the truck appointment scheduling models and directions for future research. Transp Rev 42(1):102–126

[CR3] Ambrosino D, Peirano L (2016) Truck arrival management at maritime container terminals. ECMS 2016:114–120

[CR4] Ambrosino D, Caballini C (2015) Congestion and truck service time minimization in a container terminal. In: Proceedings of the international conference on maritime-port technology and development, MTEC 2014, pp. 1–10

[CR5] Angeloudis P, Bell MG (2010) An uncertainty-aware AGV assignment algorithm for automated container terminals. Transport Res Part E: Logist Transport Rev 46(3):354–366

[CR6] Baldouski D, Dávid B, Dósa G, Dulai T, Werner-Stark A, Krész MF (2023) Managing and optimizing container flow in port logistics, pp 89–92. Slovenian Society Informatika - Section for Operational Research. https://drustvo-informatika.si/uploads/documents/6a1c2595-7d3f-4dd2-ab6c-9ed9b168c19d/SOR23Proceedings.pdf

[CR7] Bierwirth C, Meisel F (2010) A survey of berth allocation and quay crane scheduling problems in container terminals. Eur J Oper Res 202(3):615–627

[CR8] Caballini C, Gracia MD, Mar-Ortiz J, Sacone S (2020) A combined data mining-optimization approach to manage trucks operations in container terminals with the use of a tas: application to an Italian and a Mexican port. Transport Res Part E: Logist Transport Rev 142:102054

[CR9] Carlo HJ, Vis IF, Roodbergen KJ (2014) Storage yard operations in container terminals: literature overview, trends, and research directions. Eur J Oper Res 235(2):412–430

[CR10] Carlo HJ, Vis IF, Roodbergen KJ (2014) Transport operations in container terminals: literature overview, trends, research directions and classification scheme. Eur J Oper Res 236(1):1–13

[CR11] Chen G, Govindan K, Yang Z (2013) Managing truck arrivals with time windows to alleviate gate congestion at container terminals. Int J Prod Econ 141(1):179–188

[CR12] Expósito-Izquierdo C, Lalla-Ruiz E, De Armas J, Melián-batista B, Moreno-Vega JM (2017) Maritime container terminal problems. In: Handbook of heuristics, pp. 1–27. Springer, Berlin

[CR13] Gharehgozli AH, Yu Y, Koster R, Udding JT (2014) An exact method for scheduling a yard crane. Eur J Oper Res 235(2):431–447

[CR14] Gharehgozli AH, Roy D, De Koster R (2016) Sea container terminals: new technologies and OR models. Maritime Econ Logist 18:103–140

[CR15] Huynh N, Walton CM (2008) Robust scheduling of truck arrivals at marine container terminals. J Transp Eng 134(8):347–353

[CR16] Huynh N, Walton CM (2011) Improving efficiency of drayage operations at seaport container terminals through the use of an appointment system. In: Handbook of terminal planning, pp 323–344. Springer, Berlin

[CR17] Kizilay D, Eliiyi DT (2021) A comprehensive review of quay crane scheduling, yard operations and integrations thereof in container terminals. Flex Serv Manuf J 33(1):1–42

[CR18] Korsvik JE, Fagerholt K, Laporte G (2010) A tabu search heuristic for ship routing and scheduling. J Oper Res Soc 61:594–603

[CR19] Krmac E, Djordjević B (2023) Port environmental efficiency assessment using the one-stage and two-stage model DEA: comparison of Koper and Dublin ports. Environment, Development and Sustainability, pp 1–31

[CR20] MarineTraffic Live Ships Map (2024) https://www.marinetraffic.com/. Accessed 08 May 2024

[CR21] Meisel F, Bierwirth C (2013) A framework for integrated berth allocation and crane operations planning in seaport container terminals. Transp Sci 47(2):131–147

[CR22] Monaco MF, Sammarra M, Sorrentino G (2014) The terminal-oriented ship stowage planning problem. Eur J Oper Res 239(1):256–265

[CR23] Namboothiri R, Erera AL (2008) Planning local container drayage operations given a port access appointment system. Transport Res Part E: Logist Transport Rev 44(2):185–202

[CR24] Namboothiri R, Erere A (2006) A column generation heuristic for local drayage routing given a port access appointment system. In: METRANS National Urban Freight Conference, Long Beach, vol. 13. Citeseer

[CR25] Phan M-H, Kim KH (2015) Negotiating truck arrival times among trucking companies and a container terminal. Transport Res Part E: Logist Transport Rev 75:132–144

[CR26] Raeesi R, Sahebjamnia N, Mansouri SA (2023) The synergistic effect of operational research and big data analytics in greening container terminal operations: a review and future directions. Eur J Oper Res 310(3):943–973

[CR27] Stahlbock R, Voß S (2008) Operations research at container terminals: a literature update. OR Spectrum 30:1–52

[CR28] Statistical Office of the Republic of Slovenia (SURS), Port traffic (2022). https://www.stat.si/StatWeb/en/News/Index/11244. Accessed 08 May 2024

[CR29] Steenken D, Voß S, Stahlbock R (2004) Container terminal operation and operations research: a classification and literature review. OR Spectrum 26:3–49

[CR30] Stojaković M, Twrdy E (2021) Determining the optimal number of yard trucks in smaller container terminals. Eur Transp Res Rev 13:1–12

[CR31] Torkjazi M, Huynh N, Shiri S (2018) Truck appointment systems considering impact to drayage truck tours. Transport Res Part E: Logist Transport Rev 116:208–228

[CR32] Yang L, Zhang X, Li S, Luo Z (2007) The freight traffic demand forecasting of Shenzhen Yantian harbor free trade zone. Urban Transport China 2:51–54

[CR33] Yang Z, Chen G, Moodie DR (2010) Modeling road traffic demand of container consolidation in a Chinese port terminal. J Transp Eng 136(10):881–886

[CR34] Yi S, Scholz-Reiter B, Kim T, Kim KH (2019) Scheduling appointments for container truck arrivals considering their effects on congestion. Flex Serv Manuf J 31:730–762

[CR35] Zehendner E, Feillet D (2015) Benefits of a truck appointment system on the service quality of inland transport modes at a multimodal container terminal. Q Control Appl Stat 60(1):121–122

